# Comparative Transcriptome Analysis Reveals the Influence of Abscisic Acid on the Metabolism of Pigments, Ascorbic Acid and Folic Acid during Strawberry Fruit Ripening

**DOI:** 10.1371/journal.pone.0130037

**Published:** 2015-06-08

**Authors:** Dongdong Li, Li Li, Zisheng Luo, Wangshu Mou, Linchun Mao, Tiejin Ying

**Affiliations:** 1 Key Laboratory for Agro-Food Processing, College of Biosystems Engineering and Food Science, Zhejiang University, Hangzhou, People’s Republic of China; 2 Department of Postharvest Science of Fresh Produce, ARO, the Volcani Center, P.O. Box 6, Bet Dagan, Israel; South China Agricultural University, CHINA

## Abstract

A comprehensive investigation of abscisic acid (ABA) biosynthesis and its influence on other important phytochemicals is critical for understanding the versatile roles that ABA plays during strawberry fruit ripening. Using RNA-seq technology, we sampled strawberry fruit in response to ABA or nordihydroguaiaretic acid (NDGA; an ABA biosynthesis blocker) treatment during ripening and assessed the expression changes of genes involved in the metabolism of pigments, ascorbic acid (AsA) and folic acid in the receptacles. The transcriptome analysis identified a lot of genes differentially expressed in response to ABA or NDGA treatment. In particular, genes in the anthocyanin biosynthesis pathway were actively regulated by ABA, with the exception of the gene encoding cinnamate 4-hydroxylase. Chlorophyll degradation was accelerated by ABA mainly owing to the higher expression of gene encoding pheide a oxygenase. The decrease of β-carotene content was accelerated by ABA treatment and delayed by NDGA. A high negative correlation rate was found between ABA and β-carotene content, indicating the importance of the requirement for ABA synthesis during fruit ripening. In addition, evaluation on the folate biosynthetic pathway indicate that ABA might have minor function in this nutrient’s biosynthesis process, however, it might be involved in its homeostasis. Surprisingly, though AsA content accumulated during fruit ripening, expressions of genes involved in its biosynthesis in the receptacles were significantly lower in ABA-treated fruits. This transcriptome analysis expands our understanding of ABA’s role in phytochemical metabolism during strawberry fruit ripening and the regulatory mechanisms of ABA on these pathways were discussed. Our study provides a wealth of genetic information in the metabolism pathways and may be helpful for molecular manipulation in the future.

## Introduction

**Fig 1:**
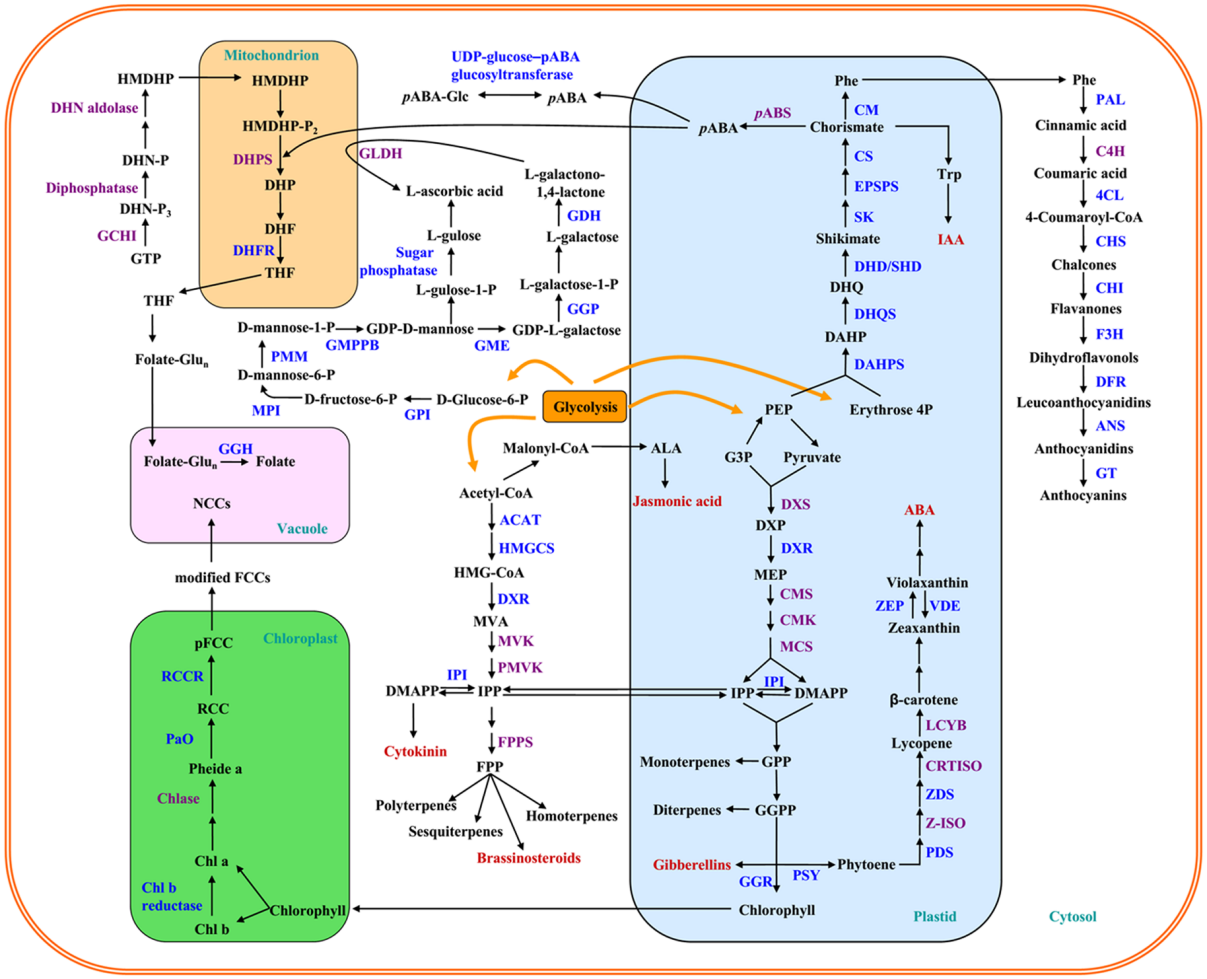
Genes identified in the metabolism network of pigments, folic acid and ascorbic acid during strawberry fruit ripening. Pathways, including anthocyanin, carotenoid, ascorbic acid and folic acid biosynthesis and chlorophyll degradation, are shown. Genes encoding enzymes with blue names were significantly differentially expressed among samples, and those encoding enzymes with purple names were identified as non-significantly differentially expressed. The chloroplast is separated from the plastid to clearly illustrate the chlorophyll breakdown pathway. Abbreviations: (anthocyanin biosynthetic pathway) 3-deoxy-7-phosphoheptulonate synthase, DAHPS; 3-dehydroquinate synthase, DHQS; 3-dehydroquinate dehydratase/shikimate dehydrogenase, DHD/SHD; Shikimate kinase, SK; 5-enolpyruvylshikimate-3-phosphate synthase, EPSPS; chorismate synthase, CS; chorismate mutase, CM; phenylalanine ammonia lyase, PAL; cinnamate 4-hydroxylase, C4H; *p*-coumarate ligase, 4CL; chalcone synthase, CHS; chalcone isomerase, CHI; flavanone 3-hydroxylase, F3H; dihydroflavonol 4-reductase, DFR; anthocyanidin synthase, ANS; glucosyltransferase, GT; (carotenoid biosynthetic pathway) 1-deoxy-D-xylulose-5-phosphate synthase, DXS; 1-deoxy-D-xylulose-5-phosphate reductoisomerase, DXR; 2-C-methyl-D-erythritol 4-phosphate cytidylyltransferase, CMS; 4-diphosphocytidyl-2-C-methyl-D-erythritol kinase, CMK; 2-C-methyl-D-erythritol 2,4-cyclodiphosphate synthase, MCS; isopentenyl-diphosphate delta-isomerase, IPI; geranyl diphosphate synthase, GGPS; geranylgeranyl reductase, GGR; phytoene synthase, PSY; phytoene desaturase, PDS; zeta-carotene isomerase, Z-ISO; zeta-carotene desaturase, ZDS; carotenoid isomerase, CRTISO; lycopene beta cyclase, LCYB; zeaxanthin epoxidase, ZEP; violaxanthin de-epoxidase, VDE; acetyl-CoA C-acetyltransferase, ACAT; hydroxymethylglutaryl-CoA synthase, HMGCS; mevalonate kinase, MVK; phosphomevalonate kinase, PMVK; farnesyl diphosphate synthase, FPPS. (Chlorophyll breakdown pathway) pheophorbide a oxygenase, PAO; red chlorophyll catabolite reductase, RCCR. (ascorbic acid biosynthetic pathway) glucose-6-phosphate isomerase, GPI; mannose-6-phosphate isomerase, MPI; phosphomannomutase, PMM; mannose-1-phosphate guanyltransferase, GMPPB; GDP-mannose 3,5-epimerase, GME; GDP-L-galactose phosphorylase, GGP; L-galactose dehydrogenase, GDH; L-galactono-1,4-lactone dehydrogenase, GLDH. (folate biosynthetic pathway) para-aminobenzoate synthase, pABS; dihydropteroate synthase, DHPS; gamma-glutamyl hydrolase, GGH. For the abbreviations of other metabolites, please refer to the manuscript.

The strawberry is one of the favorite fruits throughout the world and may be classified as a functional food as it is a rich source of phytochemicals and vitamins, both of which have relevant biological activities in human health [1,2]. The development and ripening of the strawberry fruit can commonly be divided into seven stages, and each stage is elaborately regulated by plant hormones [3]. Abscisic acid (ABA) is a phytohormone, and it has been proven to play an important role in strawberry fruit ripening, especially from the large green stage to the full red stage [4,5]. ABA is mainly synthesized from carotenoid precursors in the plastid (Fig 1) [6]. It is known that the carotenoid content is very low in strawberry fruit; however, previous studies [5,7] reported that the ABA concentration in the strawberry fruit is similar to that of the tomato fruit, a fruit typically rich in carotenoids. This suggests that carotenoids may be mainly used to synthesize ABA in strawberry fruit. Because ABA also plays important roles in many physiological processes, an investigation of ABA biosynthesis may facilitate our understanding of its functions in the regulation of strawberry fruit ripening.

During strawberry fruit development, from green to red, chlorophyll degradation is observed. Compared with researches in other species (e.g., banana and citrus fruit), the chlorophyll breakdown pathway in the strawberry fruit has not been well characterized. Chlorophyll shares a common precursor, geranylgeranyl pyrophosphate (GGPP), with carotenoids, and it is degraded in the senescent chloroplast (Fig 1). Genes involved in this pathway have not yet been identified in strawberry.

Anthocyanin is another class of pigments that is predominantly present in strawberries. The anthocyanin biosynthetic pathway is well understood, and most of the genes encoding enzymes in this pathway have been isolated and characterized from many plants (Fig 1) [8]. Anthocyanin accumulation is affected by many factors, among which ABA has been shown to enhance anthocyanin biosynthesis [9]. Previous studies in grapes showed that ABA-promoted anthocyanin synthesis might result from the transcriptional up-regulation of genes involved in the anthocyanin biosynthetic pathway [10,11]. Our previous work also revealed that exogenous ABA treatment induced anthocyanin accumulation in strawberry fruit by enhancing enzyme activities in anthocyanin biosynthetic pathway, and we proposed that cinnamate 4-hydroxylase (C4H) might not be a rate-limiting enzyme in this route [12]. However, molecular investigations on this topic are still lacking.

Folic acid and ascorbic acid (AsA) are important health-beneficial compounds in strawberry fruit, and their biosyntheses are closely related to other phytochemicals in the secondary metabolism network (Fig 1). Folate is vital for human health, unfortunately, it cannot be synthesized de novo in humans [13]. A folate deficiency can result in severe health problems, such as megaloblastic anemia [14]. Folate biofortification is an effective way to fight folate deficiency through the enhancement of folate biosynthetic precursors; however, this strategy is not always successful for all crops [15]. Therefore, further information on the folate biosynthesis pathway is needed before attempting to biofortify the strawberry fruit folate content [16]. Although strawberry is a folate-rich source, studies are mainly focused on determining the folate content and cultivar influence [17,18]. Currently, the mechanism of folate synthesis regulation is not well understood [13]. To the best of our knowledge, the effect of plant hormones on folate synthesis has not been reported. Thus, an insight into the gene transcript levels in the folate biosynthetic pathway during strawberry fruit ripening may give us a better understanding of folate biosynthesis in strawberry fruit and for future metabolic engineering.

AsA is a key player in the antioxidant system, and its content accumulates during strawberry fruit ripening [19,20]. A recent investigation of AsA synthesis in the achenes revealed that the AsA content decreased as fruit ripeness increased [21]. These results indicated that the characteristics of AsA biosynthesis in the receptacles and the achenes might be tissue-dependent. Previously, a comprehensive study of AsA biosynthesis in the whole strawberry fruit has been reported, expanding our knowledge about the regulation of this important nutrient [19]. Therefore, an examination of the AsA biosynthetic pathway in the receptacles may be helpful to further illustrate AsA’s role during strawberry fruit ripening.

Transcriptomic analysis has been widely applied in recent years to explore many scientific problems that are difficult to be solved by using other experimental methods. It may also provide us with a map for informing future studies. For example, a comprehensive transcriptomic investigation of the wild strawberry revealed that the achene ghost was more important than the embryo in plant hormones biosynthesis for fruit early stage development [22]. A transcriptome study in rice specifically found that heavy metal transportation, jasmonic acid biosynthesis and fatty acid metabolism were the three main responses to arsenic stress [23]. In this study, by taking advantage of high-throughput sequencing, we simultaneously evaluated the transcription levels of the genes involved in the metabolism of pigments including carotenoids, chlorophyll and anthocyanins, and two important vitamins during strawberry fruit ripening (Fig 1). Many genes were identified, and the regulatory role of ABA on these genes is discussed.

## Materials and Methods

### Plant material and treatments

Octaploid strawberry (*Fragaria ananassa* ‘Toyonoka’) plants were grown in a greenhouse (60%-80% relative humidity) under cycles of 14 h of light at 25°C followed by 10 h of dark at 20°C. One-hundred and forty flowers were tagged during anthesis in 2013 and 2014 (one replicate), and three replicates were created at intervals of >15 d. Two-week old fruits post-anthesis were used for pharmacological investigation. Chemical solutions of 100 μL of ABA (1 μM), the ABA biosynthesis blocker nordihydroguaiaretic acid (NDGA, 100 μM), or distilled water (used as a control), were injected with a sterile microsyringe into each fruit receptacle core through the pedicel as previously described [24]. Twenty fruits were injected with water, their achenes were removed using a scalpel, and the receptacles were immediately immersed in liquid nitrogen; these samples were named CK0 (one replicate). Based on their ripening processes, fruits were harvested on day 5 when ABA-treated fruit reached the initial red stage, a critical stage during strawberry fruit ripening [5], and on day 8, when the control fruit reached the IR stage. Achenes were removed for each treatment, the receptacles were frozen in liquid nitrogen and stored at -80°C. Correspondingly, the sample were denoted as CK5, CK8, ABA5, ABA8, NDGA5 and NDGA8.

### Total anthocyanins and chlorophyll determination

The strawberry receptacle tissue (1 g) was extracted with 5 mL of cold 1% HCl-ethanol and centrifuged at 9,000 × *g* for 15 min. The supernatants were measured for total anthocyanin content using a pH differential method [25]. The results were expressed as milligram of cyanidin 3-glucoside equivalents per 100 g of fresh weight. Total chlorophyll was extracted and determined according to the methodology of Arora et al. [26]. The experiments were repeated three times.

### β-carotene, AsA and total folate determination

To determine the β-carotene content, approximately 2 g of receptacle tissue was ground in 10 mL of cold acetone before 2 μL of dichloromethane was added. For AsA extraction, 1 g of tissue was ground in 5 mL of pre-cooled 0.1% oxalic acid. After centrifugation, the supernatant was filtered through a 0.45-μm filter. The determination of β-carotene, AsA and total folate was performed on an Agilent C18 column (250 × 4.6 mm i.d.). The mobile phases were acetonitrile:H_2_O_2_ (9:1) (solvent A) and 100% ethylacetate (solvent B) for β-carotene separation and 0.1% oxalic acid for AsA. For total folates analysis, samples were prepared according to Navattete et al. [27] and the determination of folates was performed as described previously [15]. Three biological repetitions and three technical replicas were performed for each sample.

### RNA extraction, library construction, and Illumina sequencing

Total RNA was isolated using the TRIzol reagent (Invitrogen Life Technologies, USA) following the manufacturer’s instructions and was treated with RNase-free DNase I (Takara, Japan) to avoid genomic DNA contamination. The samples were pre-treated with a Fruit-mate for RNA purification reagent (Takara) to eliminate the phenolics and sugars before RNA isolation. For each treatment and time point, RNA was extracted separately from 10 randomly selected individual fruits (as one biological replicate). Three replicates were conducted for RNA extraction. Before three biological intra-sample pooling, the individual total RNA quantity and concentration were measured on a Bioanalyzer 2100 and RNA 6000 Nano LabChip Kit (Agilent, CA, USA) with an RNA integrity number (RIN) > 8.0. mRNA was isolated from the total RNA samples using oligo (dT) attached magnetic beads (Invitrogen) and was fragmented into short pieces using divalent cations under elevated temperature. First- and second-strand cDNA was generated using mRNA-Seq sample preparation (Illumina Inc., San Diego, CA, USA) and random hexamer primers. The seven cDNA libraries underwent sequencing on an Illumina HiSeq 2000 platform, and 100-bp paired-end reads results were generated. The original data were deposited in the NCBI GEO database under accession no. GSE63224.

### Bioinformatics analysis

Low quality reads, including adaptors, low quality sequences (reads with more than 5% ambiguous bases) and reads with a Phred quality score Q ≤ 20 using the NGS QC toolkit [28], were removed from the raw reads. Unigenes were obtained by assembling the resulting clean reads with the Trinity program [29] and optimizing by TGICL [30]. Unigenes were aligned using BLASTx (E-value < 1e-5) to the NCBI non-redundant (nr) protein database. All of the unigenes were also compared with the protein databases Swiss-Prot, Kyoto Encyclopedia of Genes and Genomes (KEGG), euKaryotic Ortholog Groups (KOG) and Pfam using the BLASTx algorithm with an E-value cut-off of 1e-5. Unigene expressions were normalized with the read counts per kilobase of exon model per million reads (RPKM) method [31]. Differentially expressed genes were analyzed among groups and the plots were performed according to the method described by Yu et al. [23]. The expressions of samples at three time points in each treatment or the expressions of samples with different treatments at the same time point (see the schematic diagram of data comparison in S1 Fig) were compared by Chi-square analysis, only genes with p < 0.05 were identified as significantly differentially expressed. Deep sequencing data were further validated by quantitative reverse transcription PCR (qRT-PCR), and the primers used are listed in S3 Table. The comparison between the sequencing data and qRT-PCR results are shown in S2 Fig.

## Results

### Morphological evaluation of the whole strawberry fruit and phytochemical changes in the receptacle in response to ABA and NDGA treatment

**Fig 2:**
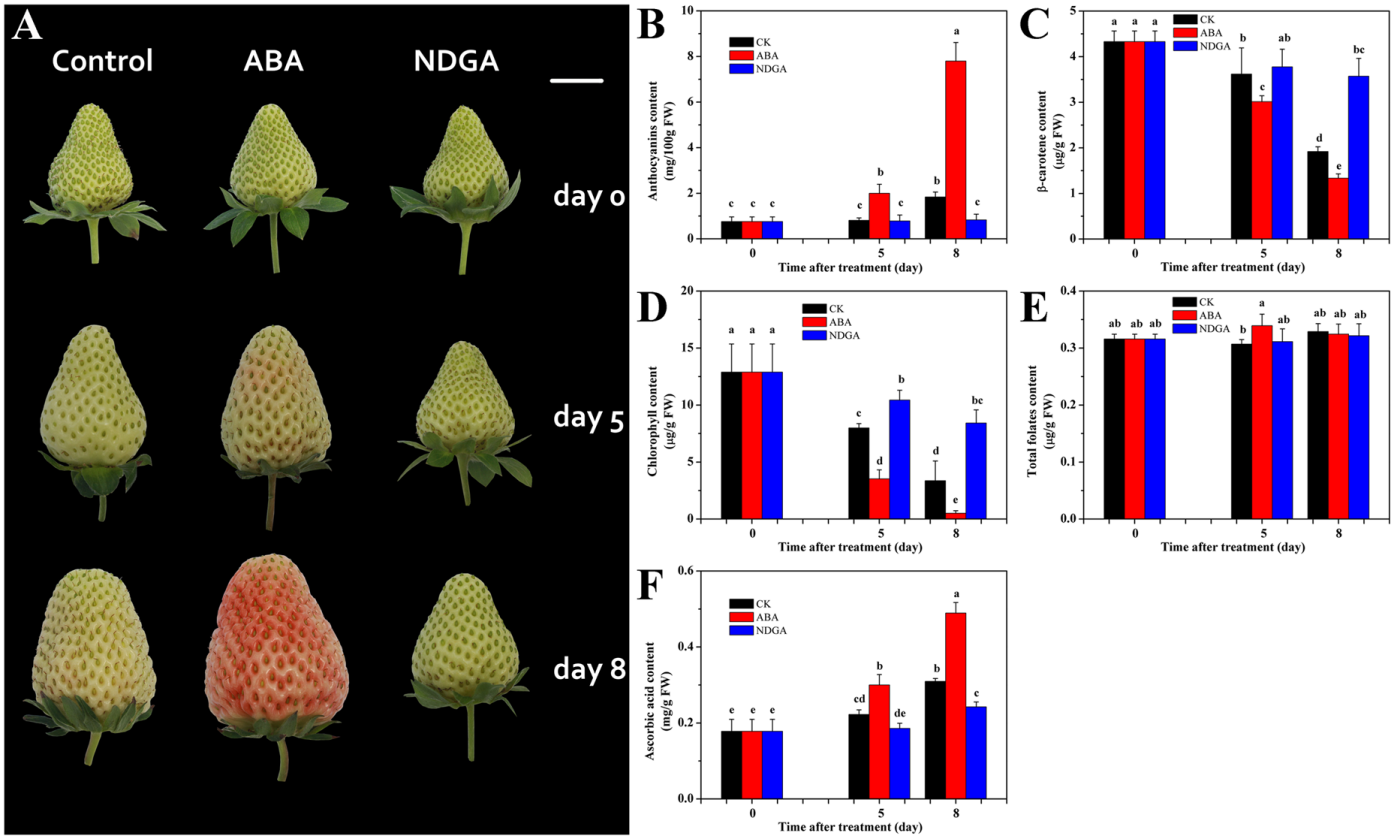
The effects of ABA and NDGA treatment on strawberry fruit ripening. (A) Morphological evaluation of the strawberry fruit in response to ABA or NDGA treatment. Changes of secondary metabolites levels in the receptacle: (B) total anthocyanin content; (C) β-carotene content; (D) chlorophyll content; (E) total folate content; and (F) ascorbic acid content. Fourteen day-old strawberry fruits after anthesis (set as day 0) were injected with chemical solutions of ABA, NDGA and distilled water for pharmacological investigation. Error bars indicate the standard deviation (SD) of means (n = 3). Bar = 1 cm. Different lowercase letters represent statistical significance (P < 0.05).

Two-week old fruits post-anthesis were injected with water, ABA, or NDGA. Until the fifth day after injection, fruits treated with ABA showed an initial red coloration (Fig 2A). ABA remarkably promoted fruit ripening until day 8; however, NDGA markedly inhibited fruit development (Fig 2A). These visual evaluations suggest that ABA plays a key role in strawberry fruit ripening and it could regulate many ripening processes.

The attractive red color of the strawberry fruit could be due to the accumulation of abundant anthocyanins during the ripening stage. The total anthocyanins content substantially accumulated during ripening in all treatments, but with different extents (Fig 2B). On day 8, compared with the control, the anthocyanins content in the ABA- and NDGA-treated fruits was 3-fold higher and 55% lower, respectively. Although the carotenoid content in strawberry fruit is low, carotenoids are the biosynthetic precusors of ABA. Therefore, it is intriguing to investigate the changes of carotenoids, such as β-carotene, in response to exogenous ABA treatment. In the present study, the β-carotene content in the strawberry receptacle decreased during fruit ripening (Fig 2C). The decreasing process was accelerated by ABA treatment and postponed by NDGA treatment. A similar pattern was observed in another important colored phytochemical, chlorophyll, which degraded while anthocyanins accumulated (Fig 2D). For nutrients of folates, no significant difference was found among the samples (Fig 2E). Meanwhile, the AsA level increased with strawberry fruit ripening. Until day 8, the AsA content in ABA- and NDGA-treated receptacles was 1.6-fold and 80% of that in the control samples (Fig 2F). In all, these results indicate that ABA may have a regulatory role in the metabolism of many phytochemicals during strawberry fruit ripening, but it has a minor influence on folate biosynthesis.

By taking advantage of high-throughput sequencing technology, we gained an insight into the specific regulatory effect of ABA on many genes involved in the secondary metabolism pathways (as listed in S1 Table) and gained a better understanding of the profile of nutrients during strawberry fruit ripening. An overview of the differentially expressed genes (those encoding enzymes labeled in blue names) in response to ABA and NDGA treatment are shown in Fig 1, and their expressions are listed in S2 Table.

### Comprehensive analysis of the genes involved in the anthocyanin biosynthetic pathway

The anthocyanin, or flavonoid biosynthetic pathway is well understood (Fig 1). Anthocyanins are synthesized in the cytosol. Preceding the anthocyanin biosynthetic pathway, phenylalanine (Phe) is synthesized from chorismate by chorismate mutase (CM). Chorismate, which is the end-product of the shikimate pathway, is also critical for *p*-aminobenzoate (*p*ABA) and indole acetic acid biosynthesis. In the phenylpropanoid pathway, Phe is catalyzed by phenylalanine ammonia lyase (PAL) to *trans*-cinnamic acid. With the catalysis of C4H and *p*-coumarate ligase (4CL), *trans*-cinnamic acid is converted to 4-coumaroyl-CoA. Chalcone synthase (CHS) is the first committed enzyme in the flavonoid pathway, and it catalyzes the conversion of 4-coumaroyl-CoA to chalcone. Chalcones are isomerized to flavanones stereospecifically by chalcone isomerase (CHI), followed by the hydroxylation to dihydroflavonols via the action of flavanone 3-hydroxylase (F3H). Thereafter, dihydroflavonols are reduced to leucoanthocyanidins by dihydroflavonol 4-reductase (DFR). Finally, leucoanthocyanidins are catalyzed by anthocyanidin synthase (ANS) to colored anthocyanidins that are modified by glucosyltransferases (GT) to anthocyanins.

**Fig 3:**
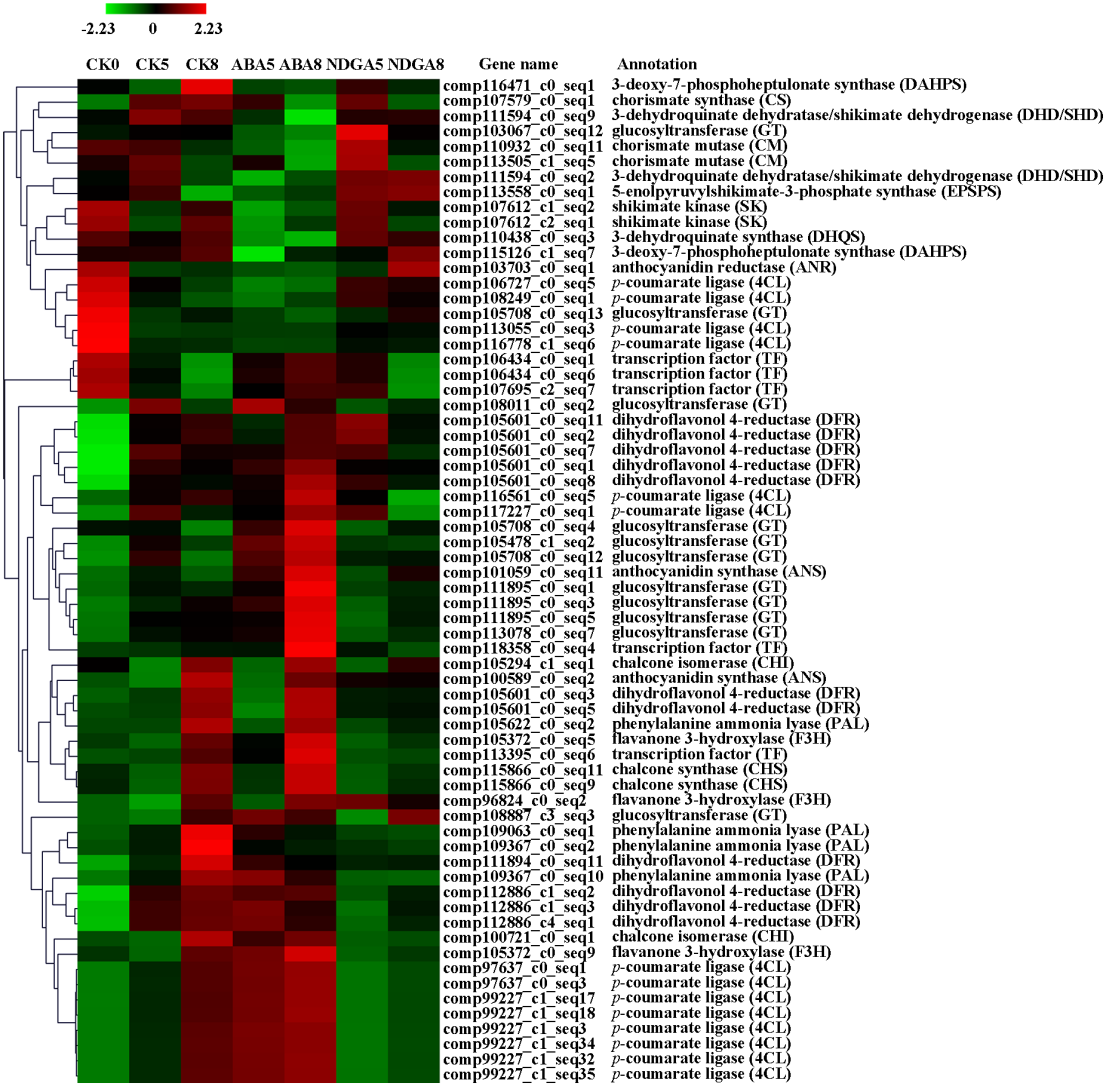
Differentially expressed genes involved in the anthocyanin biosynthesis pathway in response to ABA or NDGA treatment. CK, ABA, and NDGA represent fruit treated with water, ABA, or NDGA, respectively. Only the receptacle samples were collected for RNA-seq analysis. Red and green colors mean up- and down-regulated expression of genes, respectively. The number in each sample name represents the collection day.

Expressions of genes encoding enzymes in the anthocynin biosynthetic pathway, including the shikimate pathway, phenylpropanoid pathway and flavonoid pathway, were all significantly regulated by ABA, with the exception of *C4H* (see the blue enzyme names in Fig 1). However, most of the shikimate pathway genes showed a decreased transcript abundance during fruit ripening (Fig 3). Expressions of the genes responsible for the conversion of 3-deoxy-D-*arabino*-heptulosonate 7-phosphate (DAHP) to chorismate were lower in ABA-treated receptacles than in the controls. Delayed declines of transcriptional expressions were observed in genes such as those encoding 3-dehydroquinate synthase (DHQS), shikimate kinase (SK) and CM in NDGA-treated receptacles (Fig 3). Nevertheless, *4CL* expression was markedly up-regulated in CK8, ABA5 and ABA8 receptacles. The *CHS*, *CHI* and *F3H* genes were highly expressed in CK8 and ABA8 (Fig 3). The *GT* genes were specifically highly expressed in ABA8. In addition, one transcription factor (TF) gene (*TT8*, comp118358_c0_seq4) was also highly expressed in the ABA8 samples (Fig 3). These results revealed that all of the genes involved in anthocyanin biosynthesis actively responded to ABA or NDGA treatment, and their expressions showed a temporal and ripening degree-related changing pattern. ABA treatment might accelerate the conversion of the precursors to the final product; therefore, gene expressions in the shikimate pathway were significantly lower. However, the expression of genes encoding enzymes for the last several steps in the flavonoid pathway were remarkably higher in ABA-treated receptacles. This hypothesis could be verified from the observation of the gene expressions in NDGA-treated receptacles. Taken together, the data indicate that ABA is pivotal for the regulation of anthocyanin biosynthetic pathway gene expression, and the higher accumulation of anthocyanins content in ABA-treated receptacles may be due to the high expression of genes involved in the flavonoid pathway.

### Global analysis of the genes involved in the carotenoid biosynthetic pathway

Isopentenyl pyrophosphate (IPP) is the C_5_ isoprene unit for the start of carotenoids biosynthesis, and it is synthesized from pyruvate and glyceraldehyde-3-phosphate via the methyl-D-erythritol, 4-phosphate (MEP) pathway (Fig 1) in the plastid. IPPs are condensed to yield phytoene by phytoene synthase (PSY), with an intermediate product of geranylgeranyl pyrophosphate (GGPP), which is also a vital precursor for chlorophyll and gibberellins. Phytoene is catalyzed by a series of enzymes including phytoene desaturase (PDS), zeta-carotene isomerase (Z-ISO), zeta-carotene desaturase (ZDS) and carotenoid isomerase (CRTISO) to lycopene. Lycopene is catalyzed by lycopene β-cyclase (LCYB) to form β-carotene and further to ABA. While IPP can also be synthesized from acetyl-CoA via the mevalonic acid (MVA) pathway in the cytosol, it is important for the synthesis of phytohormone (cytokinin and brassionsteriods) and volatiles (terpenes).

**Fig 4:**
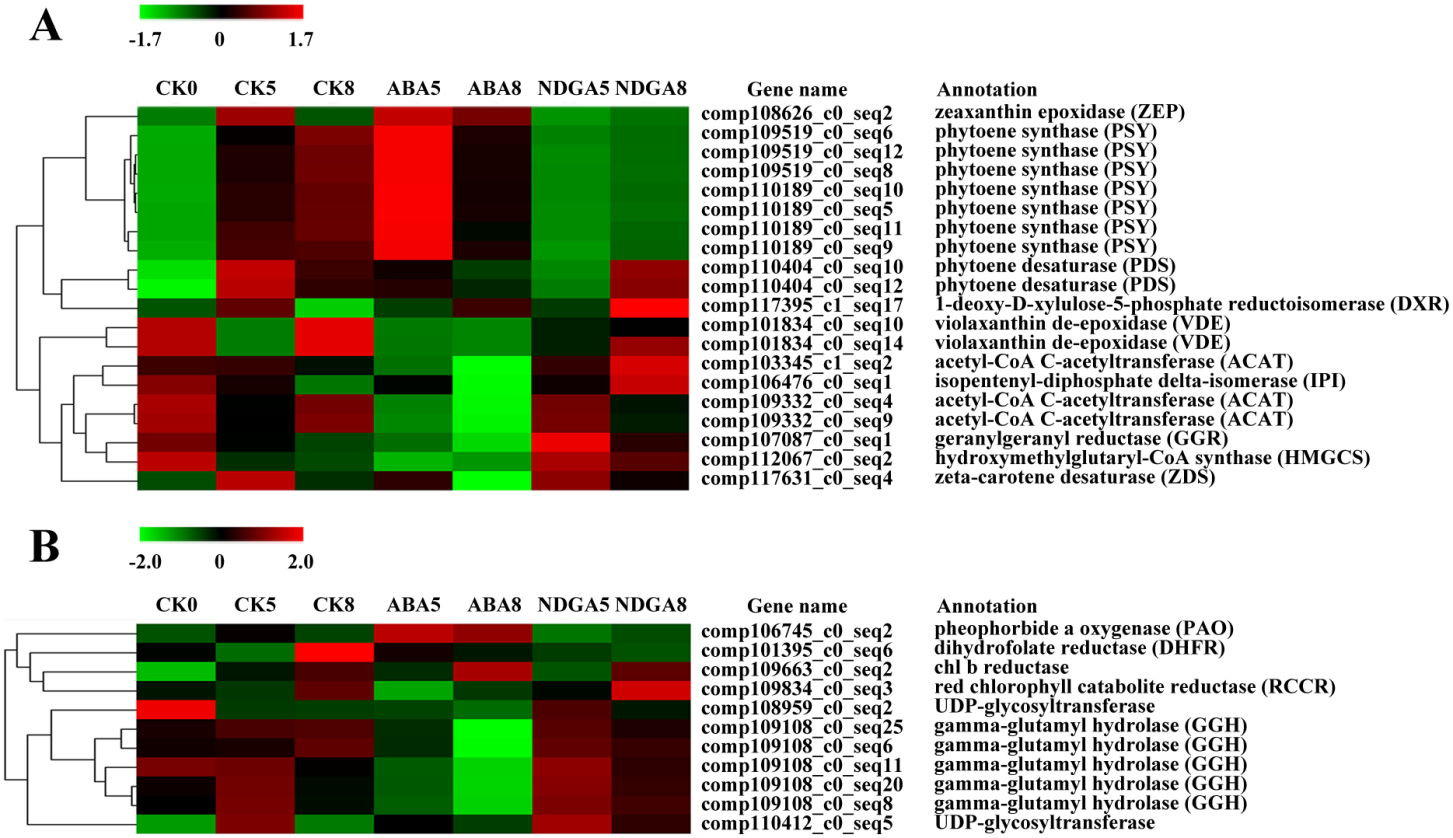
Differentially expressed genes involved in the carotenoid and folate biosynthesis pathways and the chlorophyll degradation pathway in response to ABA or NDGA treatment. (A) Gene expression in the carotenoid biosynthetic pathway. (B) Gene expression in the chlorophyll breakdown and folate biosynthetic pathways. CK, ABA, and NDGA represent fruit treated with water, ABA, or NDGA, respectively. Only the receptacle samples were collected for RNA-seq analysis. Red and green colors mean up- and down-regulated expression of genes, respectively. The number in each sample name represents the collection day.

Genes in the carotenoid biosynthetic pathway have been cloned in many other species, but have not been well identified in the strawberry. From our transcriptome data, we identified many genes that have not been reported before (such as those encoding Z-ISO and CRTISO) in this pathway, as shown in Fig 1. Genes in the primary pathway, such as *ACAT* (encoding acetyl-CoA C-acetyltransferase), *HMGCS* (encoding hydroxymethylglutaryl-CoA synthase), *DXR* (encoding 1-deoxy-D-xylulose-5-phosphate reductoisomerase) and *IPI* (encoding isopentenyl-diphosphate delta-isomerase), are highly expressed in the early ripening stage (Fig 4A). *PSY* expression was significantly up-regulated in ABA5, but decreased with fruit further ripening (ABA8). A similar transcriptional pattern was also observed in *ZEP*. As we found that ABA started to accumulate dramatically at the initial red stage (S3 Fig), the relative lower level of β-carotene in ABA-treated receptacles might be caused by the demand for ABA biosynthesis.

### Expression profiles of the genes involved in the chlorophyll degradation and the folate biosynthesis pathway

Chlorophyll(ide) b reductase (or Chl(ide) b reductase), the first enzyme for Chl b degradation, helps to convert Chl(ide) b to Chl(ide) a in the chlorophyll cycle. Chlorophyll first undergoes Mg-dechelation and dephytylation by an Mg-chelating substance (MCS) and pheophytinase (PPH), respectively. Subsequently, the product, pheophorbide a (Pheide a), is converted to a primary FCC (pFCC) by the actions of pheide a oxygenase (PAO) and red Chl catabolite reductase (RCCR). It should be noted that these two important steps are responsible for the green color loss. pFCCs are then transported from the senescent chloroplast, modified and catalyzed to produce NCCs via nonenzymatic tautomerization by the acidic conditions in the vacuole (Fig 1).

One PAO gene was observed to be markedly up-regulated by ABA treatment, whereas high expression was not found for *RCCR* (Fig 4B). Interestingly, the expression of the Chl b reductase gene was high in all three receptacle samples on day 8. From these results, we speculated that the lower chlorophyll content, as determined by spectrophotometry in ABA-treated receptacles, may be related to the higher expression of the PAO gene. The results also indicate that PAO may function as a key enzyme for chlorophyll degradation during strawberry fruit ripening. Nevertheless, genes encoding Chl b reductase and RCCR were not specifically regulated by ABA treatment.

Folate is one of the most important micronutrients in strawberry and has many forms, but only the tetrahydrofolate (THF) form has cofactor activity. THF is composed of peterin, *p*ABA and glutamate moieties. *p*ABA is synthesized from chorismate in the plastid, while peterin is derived from GTP in the cytosol; however, folate synthesis occurs in the mitochondria (Fig 1). Our results clearly showed that the expressions of genes involved in the folate biosynthesis pathway were not significantly regulated by ABA or NDGA treatment (Fig 1). Only the dihydrofolate reductase (DHFR) gene was observed to be highly expressed in CK8 and ABA5 (initial red stage). However, the genes responsible for folate homeostasis, such as gamma-glutamyl hydrolase (GGH) and UDP-glycosyltransferase, were remarkably down-regulated in ABA-treated receptacles. The results indicate that ABA has a minor influence on folate biosynthesis but it may play a regulatory role in folate homeostasis.

### Expression patterns of the genes involved in the AsA biosynthetic pathway

AsA is synthesized in the cytosol, and its precursor, D-glucose-6-phosphate, is first converted to GDP-D-mannose. Many genes involved in this pathway were identified from our sequencing data (Fig 1), including those encoding D-glucose-6-phosphate isomerase (GPI), D-mannose-6-phosphate isomerase (MPI), phosphomannomutase (PMM), and GDP-mannose pyrophosphorylase (GMPPB). There are alternative pathways for AsA synthesis from GDP-D-mannose. The first pathway is well known in plants, through L-galactose with the actions of GDP-mannose 3’,5’-epimerase (GGP), L-galactose dehydrogenase (GDH), and L-galactono-1,4-lactone dehydrogenase (GLDH). In the other pathway, AsA is synthesized through the intermediate L-gulose via sugar phosphatase, though this mechanism is not entirely clear (Fig 1).

**Fig 5:**
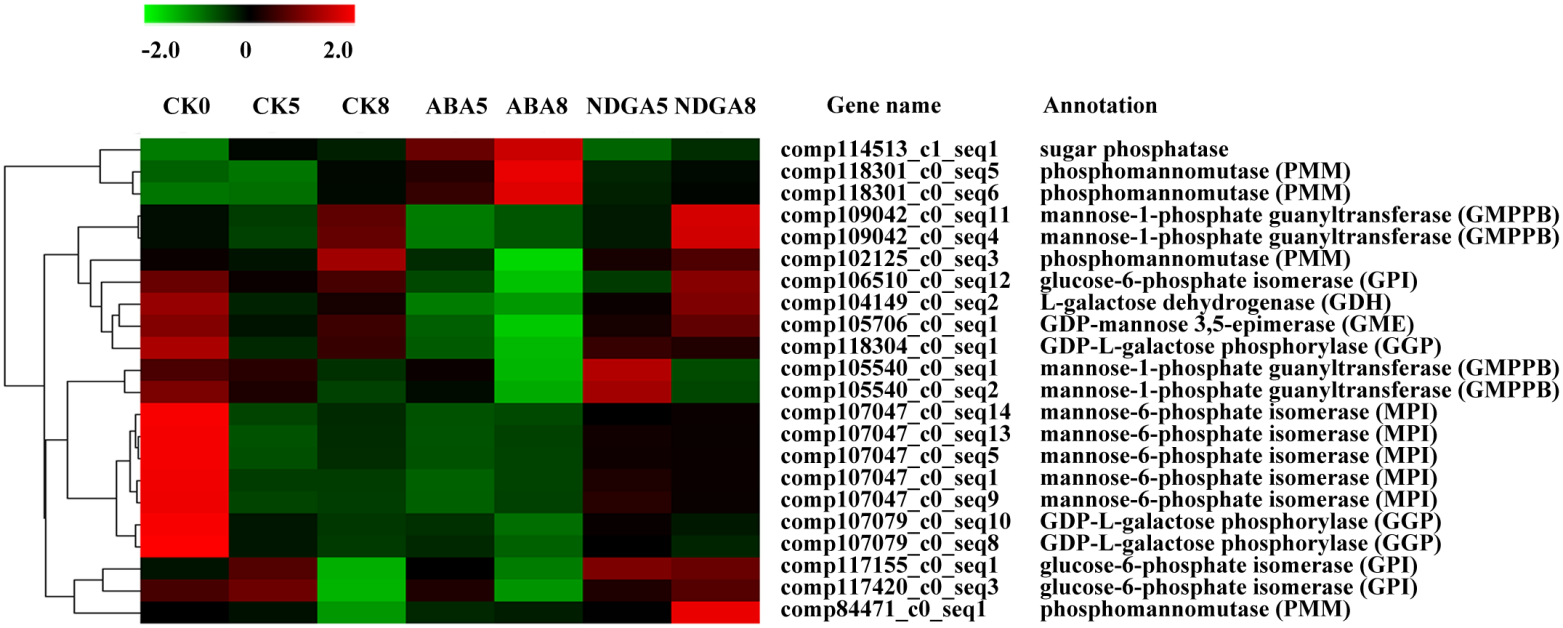
Differentially expressed genes involved in the ascorbic acid biosynthesis pathway. CK, ABA, and NDGA represent fruit treated with water, ABA, or NDGA, respectively. Only the receptacle samples were collected for RNA-seq analysis. Red and green colors mean up- and down-regulated expression of genes, respectively. The number in each sample name represents the collection day.

The *MPI* and *GGP* genes were observed to be highly expressed in CK0 receptacles (Fig 5). Much of the gene expression was significantly down-regulated by ABA treatment, such as *GPI*, *GMPPB*, *GME*, and *GDH*, whereas the expression of one gene encoding sugar phosphatase and two PPM genes (comp118301_c0_seq5 and comp118301_c0_seq6) were markedly up-regulated by ABA treatment, especially in ABA8 receptacles. No significant difference of *GLDH* expression was found among the samples, although GLDH plays a critical role for the last reduction step for AsA in the mitochondria.

## Discussion

ABA has been proven to stimulate anthocyanin biosynthesis by up-regulating the activities of enzymes or the expression of genes that are involved in the anthocyanin biosynthetic pathway in strawberry fruit [12,32]. In our study, significant differences were observed in the expressions of almost all of the genes in this pathway (Fig 1). Markedly up-regulated gene expression, especially those encoding enzymes for the later biosynthetic steps (such as ANS and GT), were found in ABA-treated receptacles. These results demonstrated the crucial regulatory role of ABA in strawberry anthocyanin biosynthesis. However, *C4H* was not differentially expressed, either in response to ABA or NDGA treatment. The *VvC4H* gene had the lowest expression levels of the anthocyanin biosynthesis related genes in grape cells, and its transcript level increased in the first 12 h after ABA treatment and then decreased back to control levels [11]. Previously, we also observed that ABA had a minor effect on C4H activity during strawberry ripening [12]. Taken together, these results indicate that *C4H* expression is transiently regulated by ABA and that C4H may not act as a rate-limiting enzyme in the anthocyanin biosynthetic pathway.

ABA plays an important role in strawberry fruit ripening, but studies pertaining to its carotenoid precursors in strawberry are scare. Our transcriptome data help us identify many genes in the carotenoid biosynthesis pathway, such as *CRTISO*, *LCYB* and *ZEP*, and these genes have not been reported before in this species. We found that the β-carotene content decreased with fruit ripening and that ABA treatment accelerated the decline (Fig 2C). The strawberry fruit is commonly regarded as a fruit containing a low carotenoids content, however, the levels of β-carotene determined in our study were similar to those in tomato fruit, as reported by Galpaz et al. [33]. We also found that the ABA content accumulated during strawberry fruit ripening and that exogenous ABA treatment stimulated its biosynthesis (S3 Fig). The correlation rate between the β-carotene and ABA content was -0.95 (S3 Fig). These results indicate the importance of carotenoids as precursors for ABA biosynthesis during strawberry fruit ripening. Previous studies in tomato indicated that an ABA deficient mutant, *hp3*, elevated the lycopene content by increasing the plastid number but not by up-regulating carotenoid biosynthesis gene transcription [33]. Another study in tomato indicated that RNAi silenced *SlNCED* increased the β-carotene content and this observation might be partially related to the action of ethylene [34]. Our data also showed a higher β-carotene content and lower expression of carotenoid biosynthesis genes in NDGA-treated receptacles (Fig 4A). Studies in rice revealed that mutations in carotenoid biosynthesis related genes reduced the ABA content [35,36]. Therefore, the higher expressions of *PSY* and *ZEP* in ABA-treated receptacles might be explained by the demand for ABA biosynthesis during fruit ripening. Our study illustrates the importance of β-carotene as an upstream compound in the strawberry ABA biosynthesis pathway.

Chlorophyll is another important colorful phytochemical in plants, and its degradation is under the control of ethylene [37]. A previous study in lichi indicated that ABA played a less important role than ethylene in chlorophyll catabolism [38]. Here, we found that ABA treatment accelerated chlorophyll degradation, while NDGA delayed the progress (Fig 2D), and higher expression levels of *PAO* were observed in ABA-treated receptacles. This indicates that the accelerating chlorophyll degradation by ABA treatment may result from the ripening processes as stimulated by ABA and *PAO* and may play a pivotal role in strawberry fruit degreening.

Previous studies have reported that ABA starts to accumulate during strawberry fruit ripening, starting from the large green stage [3,5], but the effect of ripeness was not significant for the folate content in different harvesting years [17,18]. Our results showed that the influence of ABA treatment on the folate content was minor (Fig 2E), and the expressions of all genes (except the DHFR gene) involved in folate biosynthesis were not significantly different after ABA or NDGA treatment (Fig 1). However, genes encoding the enzymes responsible for folate conjugation or homeostasis were significantly differentially expressed among the samples, and ABA down-regulated those gene expressions (Fig 4B). These results indicate that ABA might play a more important role in folate content balance than biosynthesis, and we also speculate that folate may be synthesized during early stage development. From the gene expression results during ripening, folate biofortification in the strawberry fruit may be more difficult than in other fruits (e.g. tomato) [39].

The antioxidant system functions like a variohm for reactive oxygen species (ROS) homeostasis, including ROS production and elimination, to mediate ROS perception and guide plant growth and development [40]. AsA is a key member in the antioxidant system for reactive oxygen species scavenging, especially hydrogen peroxide (H_2_O_2_). Previous studies indicated that ABA triggers H_2_O_2_ accumulation to induce an AsA content increase, thereby protecting the *Cistus albidus* plant from summer stress. Similarly, in maize seedlings, ABA was reported to enhance the AsA content to cope with oxidative stress [41,42]. In the present study, we also observed that ABA treatment enhanced AsA synthesis. This result could be explained by a higher antioxidant capacity resulting from these non-enzymatic antioxidants, which may enhance the tolerance capacity of the highly sensitive strawberry fruit to various environmental stresses. Previous studies in *Arabidopsis* and the peach indicated that AsA content had a close relationship with the transcript levels of the enzymes in its biosynthetic pathway [43,44]. However, the *GPI*, *GMPPB*, *GME*, and *GDH* genes were observed to be down-regulated as fruit ripening proceeded in this study. Similar results were also found in a comprehensive study concerning AsA and gene expression in its several biosynthetic pathways [19]. Our transcriptome data also showed a similar result as that stated by Cruz-Rus et al. [19]: no significant *GLDH* expression changes were observed among different developmental stages, although the reaction of GLDH mainly occurs in the mitochondrion (Fig 1). Moreover, these authors suggested that the abundance of different pathways varied with fruit ripeness [19], which might be applied to explain the observation of down-regulated AsA biosynthesis pathway gene expression, while AsA substantially accumulated during ripening. Nevertheless, a previous proteomic analysis of strawberry achenes reported that the AsA quantity in the green stage was significantly higher than the quantity in the red stage [21]. Considering the importance of the achenes as the main site for the biosynthesis of gibberellins and auxin [22], we speculate that AsA synthesized in the achenes could also be transported to the receptacles, and future work should be conducted on this topic.

This study examined the transcriptional profile of the influence of ABA on the metabolism of pigments and two important vitamins in strawberry, which provided a wealth of fruit ripening genomic information. Many functional genes were identified in the metabolism network, including genes involved in the carotenoid, folate and AsA biosynthesis pathways, and the RNA-seq data showed the differential gene expressions in response to exogenous ABA or NDGA treatment. This study highlights our understanding of the versatile physiological functions of ABA in the metabolism of different phytochemicals and may be helpful for molecular manipulations in the future. This study also shows that high-throughput-based transcriptome analysis is an effective way to reveal the transcriptional changes of many complex physiological processes.

## Supporting Information

S1 Fig ABA content and its correlation rate with beta-carotene. (TIF)

S2 Fig Schematic diagram of comparisons for data profiling in ABA influences on strawberry receptacle ripening. (TIF)

S3 Fig Comparison of sequencing data and qRT-PCR results. Error bars indicate the standard deviation (SD) of means (n = 3).
(TIF)

S1 Table All genes identified in the metabolism network during strawberry fruit ripening. (XLS)

S2 Table Differentially expressed genes in response to ABA or NDGA treatment. (XLS)

S3 Table Primers used in qRT-PCR experiment to validate the deep-sequencing data. (XLS)
