# Supplementary figures and images for: Comparative Transcriptome Analysis Reveals the Influence of Abscisic Acid on the Metabolism of Pigments, Ascorbic Acid and Folic Acid during Strawberry Fruit Ripening
Source: PLoS One. 2015 Jun 8;10(6):e0130037. doi: 10.1371/journal.pone.0130037 (PMC4460069; doi:10.1371/journal.pone.0130037)

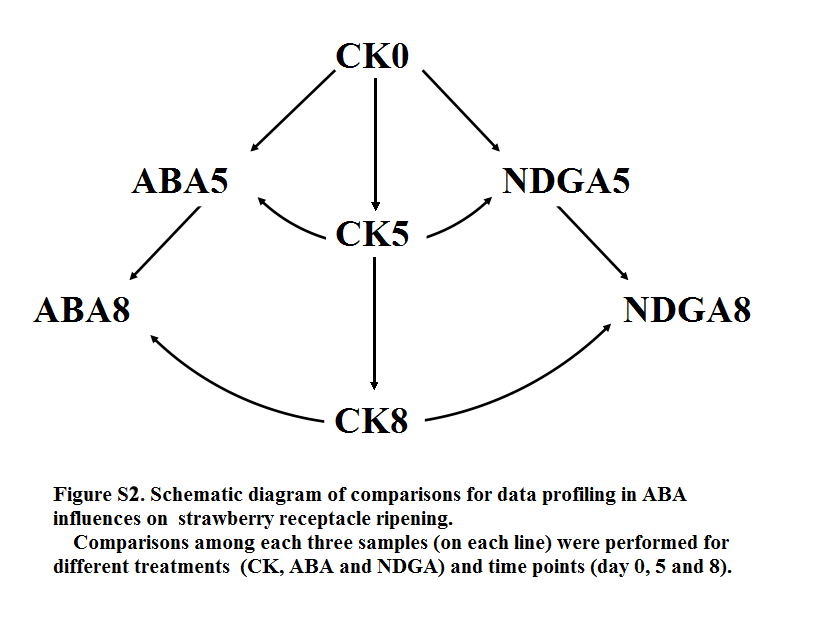

Supplement: S1 Fig — (TIF) [file pone.0130037.s001.tif]

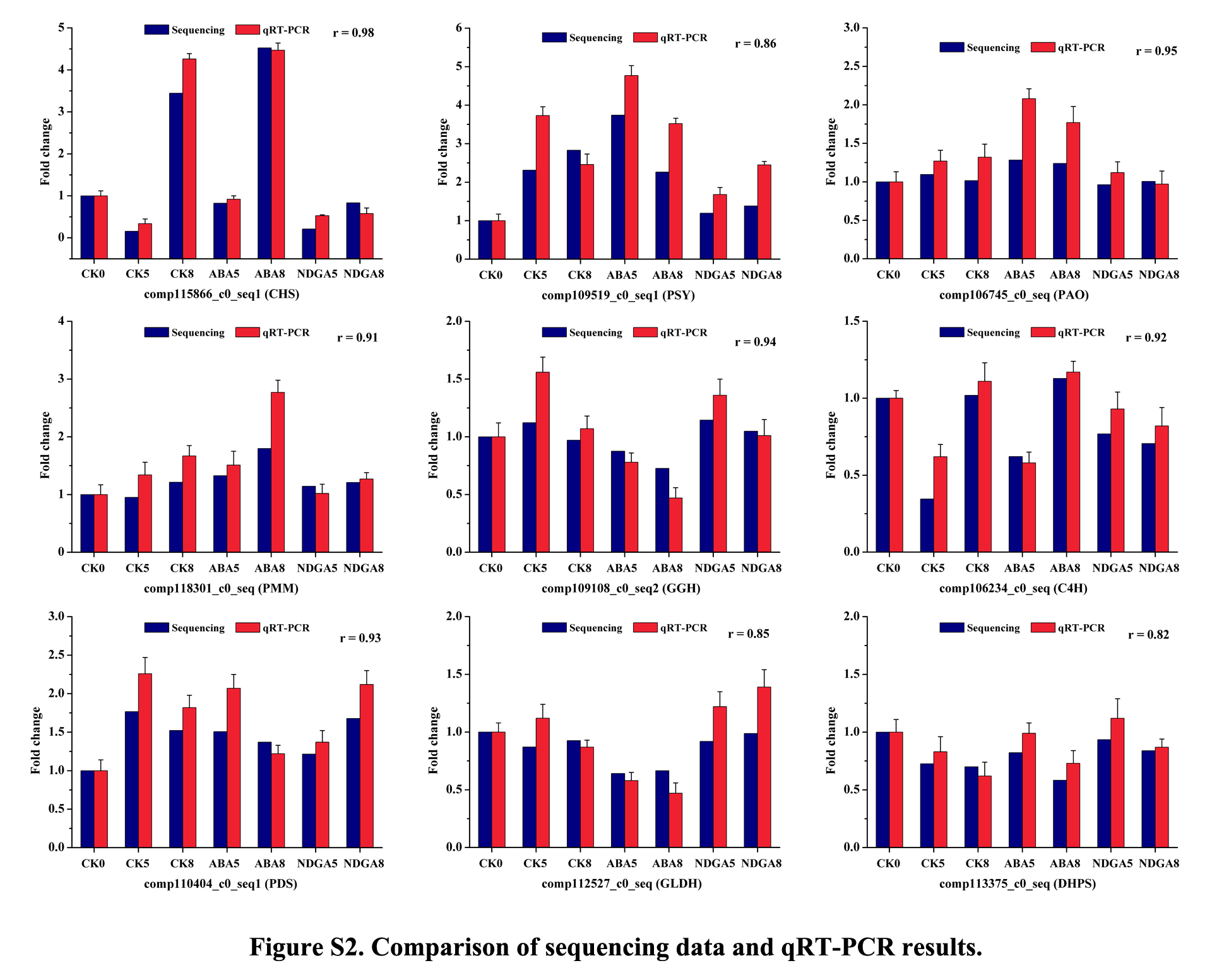

Supplement: S2 Fig — (TIF) [file pone.0130037.s002.tif]

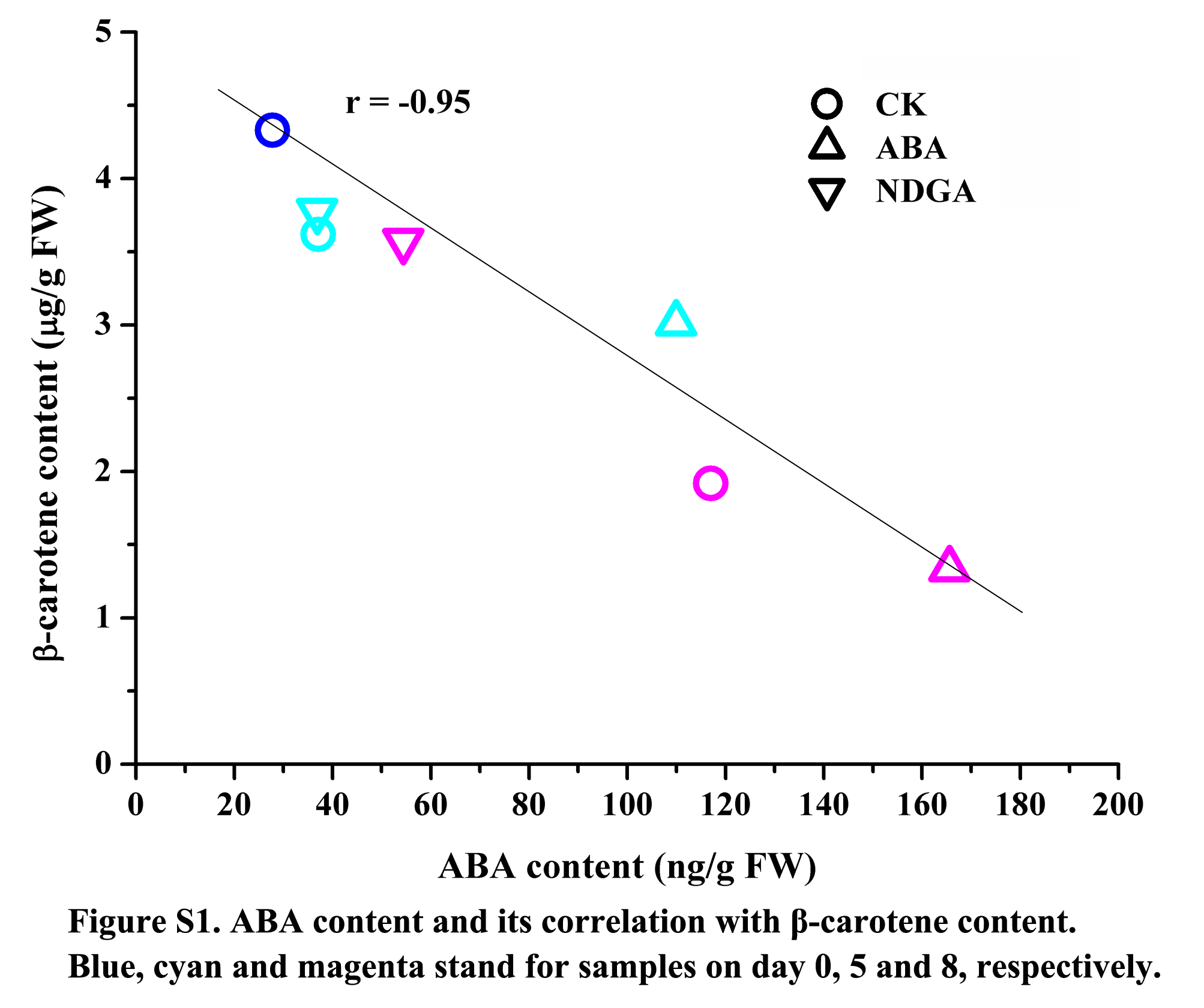

Supplement: S3 Fig — Error bars indicate the standard deviation (SD) of means (n = 3). (TIF) [file pone.0130037.s003.tif]
